# Detection of HPV and the role of p16^INK4A ^overexpression as a surrogate marker for the presence of functional HPV oncoprotein E7 in colorectal cancer

**DOI:** 10.1186/1471-2407-10-117

**Published:** 2010-03-26

**Authors:** Vanessa Deschoolmeester, Veerle Van Marck, Marc Baay, Christine Weyn, Peter Vermeulen, Eric Van Marck, Filip Lardon, Veronique Fontaine, Jan B Vermorken

**Affiliations:** 1 Laboratory of Cancer Research and Clinical Oncology, Department of Medical Oncology, University of Antwerp (UA/UZA), Wilrijk, Belgium; 2 Department of Pathology, University Hospital of Antwerp (UZA), Edegem, Belgium; 3 Research Laboratory on Human Reproduction, Université Libre de Bruxelles, Brussels, Belgium; 4 Translational Cancer Research Group, Oncology Center, General Hospital St Augustinus, Wilrijk, Belgium

## Abstract

**Background:**

Based on the well-recognized etiological role of human papillomavirus (HPV) in cervical, anogenital and oropharyngeal carcinogenesis, a potential role of HPV in colorectal carcinogenesis has been suggested. For that reason, the aim of the present study was to investigate the presence of HPV DNA in colorectal carcinomas (CRC) and to study overexpression of p16^INK4A ^as a marker for the presence of an active HPV oncoprotein E7. These findings were correlated with clinical and pathological prognostic factors of CRC.

**Methods:**

The presence of HPV was assessed using a multiplex PCR system of 10 non-biotinylated primers. The amplified fragments of HPV positive samples were further analyzed by a highly sensitive, broad spectrum SPF10 PCR and subsequently genotyped using reverse hybridization in a line probe assay.

P16^INK4A ^protein expression was investigated in a subset of 90 (30 HPV positive and 60 HPV negative) CRC samples by immunohistochemistry.

**Results:**

HPV DNA was found in 14.2% of the CRC samples with HPV16 as the most prevalent type. No significant differences in clinical and pathological variables were found between HPV positive and negative CRCs, except for age. HPV positive patients were significantly younger (p = 0.05). There was no significant correlation between the presence of HPV and overexpression of p16^INK4A ^(p = 0.325).

**Conclusions:**

In conclusion, the presence of oncogenic HPV DNA in a small cohort of CRC samples may suggest that HPV may be involved in the carcinogenesis of some CRC. However, contrary to what has been observed in head and neck squamous cell cancer and cancer of the uterine cervix, p16^INK4A ^does not seem to be a surrogate marker for an active HPV infection in CRC. Therefore, further functional analyses are necessary to elucidate the role of HPV in CRC.

## Background

Colorectal cancer (CRC) is one of the most common malignancies throughout the Western World. Surgery is the cornerstone in the treatment of patients with CRC and is followed by adjuvant chemotherapy and radiotherapy for specific subgroups of patients [1]. Although many risk factors for development of CRC have been identified, the molecular mechanisms related to the colorectal carcinogenesis remain to be elucidated [2]. Already, for quite some time studies have given evidence for an association of human papillomavirus (HPV) and CRC [2-12]. Based on the well-recognized etiologic role of HPV in cervical, anogenital and oropharyngeal carcinogenesis, a potential role of HPV in colorectal carcinogenesis has been suggested. However, so far the outcome of studies investigating this provided contradictory results [2-12]. Many authors [2-5,7,8,10-12] were able to detect HPV DNA in CRC by different laboratory techniques, but others failed to demonstrate its presence [9,13-16]. Despite the eventual presence of HPV DNA in CRC, it has remained uncertain whether HPV is simply a casual passenger or whether it has a causal role in colorectal carcinogenesis. Apart from a general contribution to fully understand the biology of this disease, such a causal role of HPV in colorectal carcinogenesis could have important implications in patient care and colorectal cancer prevention.

The HPV viral oncogenes E6 and E7 have shown to be the main contributors to the development of HPV induced cancers. These oncogenes have the ability to bind host cell regulatory proteins, especially tumor suppressor gene products [17]. The HPV oncoprotein E7 is known to bind and inactivate hypophosphorylated retinoblastoma protein (pRB) [18], which eventually leads to upregulation of p16^INK4A^. P16^INK4A ^is a tumor suppressor protein that inhibits cyclin dependant kinases (CDK)-4 or -6 binding to cyclin D which regulates the G1 cell cycle checkpoints [19,20]. Overexpression of p16^INK4A ^is considered to be strong and consistent in HPV-induced cancers [21]. Therefore, overexpression of p16^INK4A^, as detected by immunohistochemistry, has shown to be a useful adjunct to cytology in cervical cancer screening [22], a reliable marker of human papillomavirus-induced oral high-grade squamous dysplasia [23], and a useful adjunct in the assessment of biopsies for HPV-associated anal intraepithelial neoplasia [24]. Furthermore, in primary rectal squamous cell carcinoma (SCC) there was a clear association between strong reactivity for p16^INK4A ^and the presence of high-risk HPV [25]. However, that study was limited to three patients.

The aim of the present study was to investigate the presence of HPV DNA in a series of colorectal carcinomas. In a second part of the study, overexpression of p16^INK4A ^was investigated as a marker for the presence of an active HPV oncoprotein E7 in a subset of the above mentioned series of colorectal cancers. Subsequently, the results were analyzed for correlation with prognostic clinical features for disease outcome and pathological variables.

## Methods

### 1. Tissue samples

Material from a previous study of patients with CRC treated at the Antwerp University Hospital in Edegem or the St. Augustinus Hospital in Wilrijk [26] was used for HPV detection as described below. A total of 232 CRC samples were eligible for HPV detection. This comprised 90 females and 142 males with a median age of 59.4 years (range 30 to 88 years). TNM staging was determined and the distribution was as follows: 27 patients were classified as stage I (12.2%), 68 as stage II (30.6%), 74 as stage III (33.3%) and 53 as stage IV (23.9%). Seventy patients had a tumor located in the proximal region of the colon (30.2%), while 80 tumors were found in the distal colon (34.5%) and 71 in the rectum (30.6%).

All HPV positive tumors, except three (n = 30), plus two randomly chosen HPV negative tumors per HPV positive tumor (n = 60) were used for p16^INK4A ^immunohistochemistry. Three HPV positive samples could not be investigated by immunohistochemistry since the paraffin blocks were no longer available. The study was approved by the local Ethics Committee of the University of Antwerp and was conducted in accordance with the ethical principles stated in the most recent version of the Declaration of Helsinki.

### 2. DNA isolation

Tumor DNA was obtained from formalin-fixed, paraffin embedded tissue blocks. After manual microdissection to enrich for tumor cells, DNA was isolated as described previously [27]. After DNA extraction, adequate DNA isolation was confirmed by β-globin PCR [28], generating a fragment of 110 bp.

### 3. PCR and genotyping analysis of HPV

Since formalin-fixed paraffin embedded materials often yield poorly amplifiable DNA, the efficacy of the primer pair is inversely correlated with the length of the amplimers and the primers should be designed to amplify a relatively short PCR fragment [29]. DNA samples were first tested in a genital HPV broad spectrum PCR using 10 non-biotinylated short PCR fragment (SPF) primers. The SPF primers sets are designed to amplify a 65 bp fragment located within the L1 region of HPV [30,31] allowing highly sensitive detection of HPV DNA. PCR reactions were performed in a final volume of 50 μl containing 1.25 units of iTaq DNA polymerase (BioRad, Nazareth, Belgium), 2 mM MgCl_2_, 200 μM deoxynucleotide triphosphate, 1× iTaq buffer, 15 pmol of each of the forward and reverse primers and 10 μl of isolated DNA. The PCR reactions were carried out using the iCycler (BioRad) as previously described [32], except that activation of the enzyme was carried out for 3 min at 95°C. Each experiment was performed with separate positive (1 pg and 10 pg HPV16 stable SiHa cells) and negative PCR controls. After analysis on ethidiumbromide stained agarose gel analysis, positive samples were re-amplified using biotinylated SPF_10 _primers (InnoGenetics, Ghent, Belgium).

HPV genotyping was performed using reverse hybridization by the INNO-line probe assay (INNO-LiPA, Innogenetics) as described earlier [30]. The current version of the SPF_10 _LiPA contains probes for high-risk HPV genotypes 16, 18, 31, 33, 35, 39, 45, 51, 52, 56, 58, 59, 66, 68 and 70, and low risk HPV genotypes 6, 11, 34, 40, 42-44, 53, 54 and 74. The hybridization steps were carried out following the INNO-LiPA kit's instruction. Briefly, oligonucleotide probes (containing a poly d(T) tail) were immobilized in parallel lines on nitrocellulose membrane strips by the supplier; 10 μl of the PCR product, containing biotin at the 5' end of the primers, was denaturated by adding 10 μl of NaOH solution. After hybridization of the PCR product to the probes on the strip under stringent conditions, followed by stringent washing, the hybrids were detected by alkaline phosphatase/streptavidin conjugate and subtrate (5-bromo-4-chloro-3-indolylphosphate and nitroblue tetrazolium), resulting in a purple precipitate at the positive probe lines. After drying, the strips were interpreted visually by using the INNO-LiPA HPV genotyping v2 interpretation chart [33].

### 4. Immunohistochemistry

Five μm-thick sections were prepared from formalin-fixed paraffin-embedded tissue for IHC. Sections were deparaffinized in toluene, dehydrated and subjected to heat antigen retrieval in Epitope retrieval solution (as supplied in the CINtec Histology Kit, mtm laboratories, Heidelberg, Germany) in a heating bath for 30 min. at 95 (± 1)°C.

Sections were subsequently stained using the CINtec Histology Kit (mtm laboratories) on a Dako Autostainer Plus system (DAKO, DakoCytomation, Glostrup, Denmark). Endogenous peroxidase activity was quenched by incubating the slides in peroxidase blocking reagent for 10 minutes. Incubation with mouse anti-human p16^INK4A ^monoclonal antibody (diluted 1:100) was performed for 30 minutes at room temperature. Sites of binding were detected using 3,3'-diaminobenzidine (DAB^+^) as chromogen according to the manufacturers instructions. The sections were counterstained with haematoxylin, dehydrated, cleared and mounted.

### 5. MSI analysis

All cases had been previously analyzed for MSI status [26]. After manual microdissection of formalin-fixed, paraffin embedded tissue blocks, DNA was isolated as described previously [27]. MSI analysis was performed using the mononucleotide multiplex system as described earlier [34]. In short, the sense primers were chemically labeled at the 5' end with FAM™ fluorescent dyes. PCR was carried out in a final volume of 25 μl containing 200 μmol/L dNTPs (MBI Fermentas, St. Leon-Rot, Germany), 500 nM of each sense and antisense primer (Eurogentec, Seraing, Belgium), 1 × PCR buffer (60 mM Tris SO_4 _(pH 8.9), 18 mM (NH_4_)SO_4 _and 2 mM MgSO_4_) and 1 unit Discoverase dHPLC DNA polymerase (Invitrogen, Merelbeke, Belgium). Fluorescent PCR products were analyzed by capillary electrophoresis using an ABI 3100 Genetic Analyzer (Applied Biosystems, Lennik, Belgium) and Genemapper Software 3.7.

### 6. Statistics

Prognostic relevance of HPV was assessed by survival analysis. Survival probability was estimated using the Kaplan and Meier method. Differences were tested using the log rank statistic. The median follow up for OS and DFS was 4.5 and 3.7 years respectively for the entire study population and 5.8 and 4.8 years respectively for the subpopulation of 90 colorectal tumors used for p16^INK4A ^IHC.

Possible associations between the presence of HPV-DNA and clinicopathological parameters of colorectal cancers were investigated using the χ^2^-test or Fisher's exact test (when appropriate) for categorical variables and using Student t-test or Mann-Whitney U test (when appropriate) for continuous variables. In order to assess the independent prognostic contribution of HPV, a multiple Cox regression analysis was conducted. All analyses were conducted using SPSS (version 16.0). Significance for all statistics was two-tailed and recorded if p < 0.05.

## Results

### 1. HPV detection and genotyping

All tissue samples were positive for the β-globin gene, indicating that DNA was available for molecular analysis. HPV DNA was detected in colorectal tissue in 33 out of 232 patients (14.2%) using SPF_10 _PCR.

**Figure 1:**
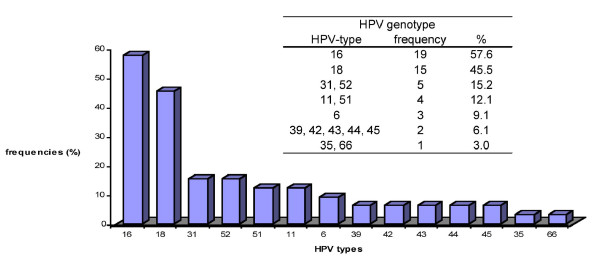
**Frequencies of different HPV-types detected in HPV-DNA positive CRC tissue samples**.

HPV DNA-positive samples were subsequently genotyped using the SPF_10 _LiPA by reverse hybridization (Innogenetics). In about half of the samples a single HPV infection was identified (54.5%) whereas the other HPV-DNA positive samples contained multiple HPV infections (45.5%). A relative broad spectrum of HPV genotypes was found, HPV 16 (57.6%) being the most prevalent type, followed by HPV 18 (45.5%) (Figure 1). The low risk HPV types 6, 11, 42, 43 and 44 were also found in a limited number of CRC samples, but, with one exception (for HPV type 43), always in the presence of a high risk HPV type.

### 2. Correlations of HPV with clinicopathological variables and survival

The median age of the overall population is 59 years. HPV positive patients were younger (median age: 56 years) than HPV negative patients (median age: 60 years) but the difference was of borderline significance (p = 0.05). Anatomic location of the tumor had no correlation with the presence of HPV infection. HPV prevalence was similar in proximal colon, distal colon, and rectum (p = 0.565). The location of the tumors throughout the colon in correlation to the presence of HPV is shown in Table 1.

**Table 1:** Location of the tumors throughout the colon in correlation to the presence of HPV.

	HPV positivity	n	%	% total
Proximal	caecum	6	18.2	39.4
	hepatic flexture	3	9.1	
	colon ascendens	1	3.0	
	right hemicolon	3	9.1	
Distal	rectosigmoid	4	12.1	30.3
	sigmoid	5	15.2	
	colon descendens/sigmoid	1	3.0	
Rectum		10	30.3	30.3

N = number of HPV positive samples, % = percentage of HPV positive samples per location separately, total %: percentage of HPV positive samples in proximal, distal and rectal tumors.

Clinical and pathological features were studied between HPV positive and negative carcinomas. The results are shown in Table 2, with no significant difference being observed. In addition, follow-up for overall survival (OS) and disease free survival (DFS) was available for 220 and 194 colorectal cancer patients, respectively. At the end of the observation period, 86 of the 220 (39.3%) patients had died while 72 of the 194 (37.3%) had experienced a recurrence of the tumor. All deaths were tumor related. No statistically significant difference in OS (HR: 0.73, p = 0.35) and DFS (HR: 0.84, p = 0.61) was found between HPV positive and HPV negative CRC patients in univariate analysis.

**Table 2:** Possible associations between HPV and clinicopathological parameters of colon cancer

		-	HPV %	+	%	
MSI	MSS	187	94.4	31	93.9	1
	MSI	11	5.6	2	6.1	
						
age (median)		56		60		**0.05**
						
gender	male	119	59.8	23	69.7	0.337
	female	80	40.2	10	30.3	
						
grade of differentiation	good	83	44.1	12	40.0	0.764
	average	87	46.8	16	53.3	
	poor	17	9.0	2	6.7	
						
location	proximal	57	30.3	13	39.4	0.565
	distal	70	37.2	10	30.3	
	rectum	61	32.4	10	30.3	
						
lymph nodes	+	97	86.6	82	89.1	0.670
	-	15	13.4	10	10.9	
						
stage	I	25	13.0	3	9.7	0.265
	II	55	28.7	13	41.9	
	III	62	32.3	12	38.7	
	IV	50	26.0	3	9.7	
						
adjuvant	yes	62	37.8	22	73.3	0.303
	no	102	62.2	8	26.7	

Possible associations were investigated using the χ^2^-test or Fisher's exact test (when appropriate) for categorical variables and using Student t-test or Mann-Whitney U test (when appropriate) for continuous variables (MSI: microsatellite instability, MSS: microsatellite stability)

### 3. P16^INK4A ^expression in CRC

**Figure 2:**
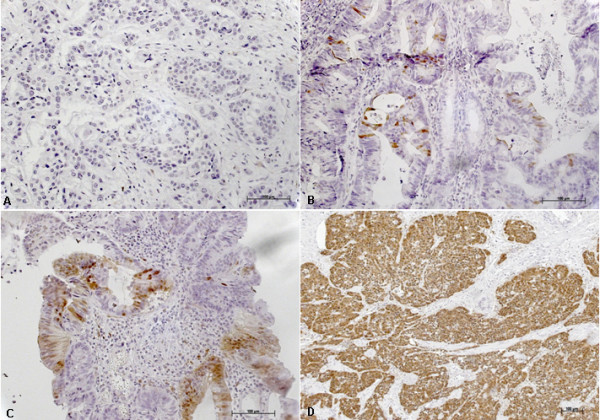
**Immunohistochemical analysis of p16^INK4A ^expression in colorectal carcinomas**. A: no p16^INK4A ^expression (negative) in tumor cells. B: weak expression of p16 in tumor cells. C: moderate expression of p16^INK4A ^in tumor cells and D: strong expression of p16^INK4A ^with a strong intensity in tumor cells.

P16^INK4A ^stained slides (n = 90) were scored on two separate aspects: the number of P16^INK4A ^positive tumor cells present (0: <5%; 1: 5-25%; 2: 25-50%; 3: > 50%), and the intensity of p16^INK4A ^staining (0 = absent; 1 = weak; 2 = moderate; 3 = strong). Some examples of the different scores are shown in Figure 2. After the development of this scoring system, 57 slides were scored again after a two month interval, to assess the reproducibility of the scoring system. Although some differences were noted, both in cell numbers and in intensity, the reproducibility of the scoring system was high (Kappa: 0.831 and 0.742 for cell numbers and intensity respectively). Both aspects were subsequently weighed to come to a final score as shown in Table 3, again, reproducibility after a two month interval was very high (Kappa: 0.975). Seventy-four percent (n = 67) of all CRC tumors showed p16 expression ranging from weak (n = 11) over moderate (n = 18) to strong (n = 38). The results of p16^INK4A ^IHC in relation to presence or absence of HPV are given in Table 4. It is obvious from these results that there is no significant correlation between the presence of HPV and overexpression of p16^INK4A ^(p = 0.325) in the colorectal cancer tissues examined.

**Table 3:** Scoring system for P16^INK4A^

Intensity				
# cells	0	1	2	3
0	negative	negative	negative	negative
1		weak	weak	moderate
2		weak	moderate	strong
3			strong	strong

**Table 4:** Possible associations between p16^INK4A ^expression and clinicopathological parameters of colon cancer

p16 expression										
		neg	%	weak	%	mod	%	strong	%	P
HPV	-	14	60.9	10	90.9	12	66.7	24	63.2	0.325
	+	9	39.1	1	9.1	6	33.3	14	36.8	
										
MSI	MSS	19	86.4	11	100.0	17	94.4	37	97.4	0.266
	MSI	3	22.6	0	0.0	1	5.6	1	2.6	
										
age (median)		63		70		66		65		0.344
										
gender	male	10	43.5	7	63.6	12	66.7	21	55.3	0.466
	female	13	56.5	4	36.4	6	33.3	17	44.7	
										
lymph nodes	-	9	60.0	6	66.7	6	66.7	24	68.6	0.951
	+	6	40.0	3	33.3	12	33.3	11	31.4	
										
grade of differentiation	good	12	54.5	7	63.6	8	44.4	18	51.4	0.875
	moderate	7	31.8	3	27.3	9	50.0	13	37.1	
	poor	3	13.6	1	9.1	1	6.6	4	11.4	
										
location	proximal	16	69.6	2	18.2	4	22.2	8	22.2	0.002
	distal	2	8.7	3	27.3	9	50.0	16	44.4	
	rectum	5	21.7	6	54.5	5	27.8	12	33.3	
										
stage	I	3	13.0	4	40.0	2	11.8	4	10.8	0.066
	II	8	34.8	2	20.0	4	23.5	18	48.6	
	III	10	43.5	4	40.0	6	35.3	13	35.1	
	IV	2	8.7	0	0.0	5	29.4	2	5.4	
										
adjuvant therapy	no	6	30.0	6	75.0	5	33.3	15	55.6	0.186
	yes	14	70.0	2	25.0	10	66.7	12	44.4	

Possible associations were investigated using the χ^2^-test or Fisher's exact test (when appropriate) for categorical variables and using Student t-test or Mann-Whitney U test (when appropriate) for continuous variables (MSI: microsatellite instability, MSS: microsatellite stability).

### 4. Correlation of p16^INK4A ^expression with clinicopathological variables and survival

Anatomic location of the tumor showed a significant correlation with the overexpression of p16^INK4A^. Tissues obtained from the proximal colon showed significantly less expression of p16^INK4A ^compared to tissues taken from the distal colon and the rectum (p = 0.002). The location of the tumors throughout the colon in correlation to the p16^INK4A ^expression is shown in Table 5. There was also a trend towards a correlation between p16^INK4A ^expression level and stage (p = 0.066). Clinical and pathological features were studied between carcinomas with and without expression of p16^INK4A^. The results are shown in Table 4, with no significant differences being observed.

**Table 5:** The location of the tumors throughout the colon in correlation to the p16^INK4A ^expression

p16 expression									
		negative		weak		moderate		strong	
proximal	right hemicolon	7	30.4%	2	18.2%	2	11.1%	2	5.6%
	caecum	3	13.0%	0	0.0%	1	5.6%	3	8.3%
	colon asecendens	1	4.3%	0	0.0%	0	0.0%	1	2.8%
	colon transversum	1	4.3%	0	0.0%	0	0.0%	1	2.8%
	hepatic angle	4	17.4%	0	0.0%	0	0.0%	1	2.8%
	jejenum	0	0.0%	0	0.0%	1	5.6%	0	0.0%
									
distal	left hemicolon	0	0.0%	0	0.0%	1	5.6%	3	8.3%
	recto-sigmoid	1	4.3%	0	0.0%	2	11.1%	1	2.8%
	sigmoid	1	4.3%	3	27.3%	6	33.3%	12	33.3%
									
rectal	rectum	5	21.7%	6	54.5%	5	27.8%	12	33.3%
									
	TOTAL	23	100.0%	11	100.0%	18	100.0%	36	100.0%

The actual location of the tumor in the large bowel could not be retrieved in 2 cases.

In addition, follow-up for overall survival (OS) and disease free survival (DFS) was available for 88 and 86 colon cancer patients, respectively. At the end of the observation period, 37 (42%) patients had died while 31 (36%) had experienced a recurrence of the tumor. All deaths were tumor related. No statistically significant difference in OS (HR: 1.06, p = 0.70) and DFS (HR: 0.96, p = 0.76) was found between CRC patients with and without p16^INK4A ^expression.

## Discussion

Oncogenic papillomaviruses have shown to be involved in benign and malignant lesions of the cervix and other anogenital sites [10]. Although the squamous cell epithelium is the most frequent target site of human papillomavirus (HPV) infection, similar infections have been demonstrated in other neoplasms, including adenocarcinomas of the cervix [3].

Based on its well-known role in cervical and anogenital carcinogenesis, some studies proposed an association between HPV and CRC [2-12]. DNA viruses are known to activate proto-oncogenes (p53, pRB and c-myc...) [3] and a collaboration of the ras oncogene with HPV E6/E7 genes inducing full transformation of normal cells has been suggested by several groups [12,35-38].

The presence of HPV DNA in colonic neoplasms is a conflicting issue. Although earlier studies have failed to detect HPV DNA in colon biopsy samples [13,14], more recent reports have suggested that infection with HPV16 and 18 may be etiologically associated with some cases of CRC [2-5,7,8,10-12,39-41]. In the present study, HPV DNA was found in 14.2% of CRC. Single infections as well as multiple infections were present and all positive samples, except one, contained at least one high-risk HPV type. The HPV frequency is lower than that found in previous studies where HPV was detected in 21.9 - 97% of CRC samples [2-5,7,8,10-12,39-41]. The discrepant results might be attributed to methodological differences (for instance the use of L1 versus E6/7 primers sets) among the studies, or differences in sensitivity of the methods used for the analysis (for instance due to differences in amplicon length, since it has been shown that the efficiency of the primer pair is inversely correlated to the length of the amplicon in formalin fixed paraffin embedded tissues [29]). In addition, regional variations in the prevalence of HPV infection, which is known to be influenced by the ethnical and geographical origin of the individuals being tested, might also contribute to the differences observed among published studies [2,7]. However, we took the necessary precautions (during microdissections, DNA extractions and PCR reagents preparations) to avoid cross-contamination and the SPF_10 _PCR is proven to be a very sensitive HPV detection technique [30,31]. Modes of transmission of HPV infection in the colon region have not been fully resolved; however, anal transmission and an association between sexual behavior and risk for HPV-positive cancers have been suggested [10]. In accordance to Bodaghi et al. [11] and Damin et al [2], there was no significant difference in the distribution of the virus throughout the colon (p = 0.565). Rates of viral detection were similar in tissues taken from the proximal colon, the distal colon or the rectum, suggesting that HPV is not a result of retrograde viral transmission from the anogenital area [2]. One possible hypothesis could be that during a screening colonoscopy, an anal HPV infection might be transported from the anal region throughout the colon [42]. Likewise, it has been shown that HPV DNA can be present on specula, used for taking PAP smears, and autoclave sterilization is the method of choice to eradicate these viruses [41,43]. Transfer by colonoscopy might also explain the lower rate of HPV infection in our study population because screening for CRC is much less common in Belgium than in the US. However, considering that HPV infection is mainly transmitted by cell surface contact, the route of viral transmission to the colon remains to be determined [2].

As seen in most other studies, high-risk HPV type 16 was the most prevalent type in colorectal tissues in this study, followed by high-risk HPV type 18. These types have been reported to suppress tumor suppressor proteins functions and play an important part in carcinogenesis [7]. Low risk types were also detected in CRC but, with one exception, always along with the presence of a high risk type. However, in order to suggest that HPV might be involved in colon cancer carcinogenesis, viral DNA incorporation into the host genome needs to be demonstrated by in situ hybridization. In addition, the presence or absence of HPV in a non-malignant control group and tumor adjacent tissue needs to be investigated in order to determine whether HPV is merely an epiphenomenon in CRC or rather a potential cofactor in the development of the disease [2].

No significant differences in clinical and pathological variables were found between HPV positive and negative colorectal carcinomas. HPV positive patients showed a trend to be younger than HPV negative patients. A similar observation has been made in HPV positive oral squamous cell cancer, a disease in which HPV appears to play an etiologic role [44-46]. Other reports in CRC have failed to demonstrate a correlation between the presence of HPV and prognostic factors [2,3,5,7,8,10-12]

In the second part of the study, overexpression of p16^INK4A ^was investigated as a marker for the presence of an active HPV oncoprotein E7 in a subpopulation of colorectal carcinomas. It has been demonstrated that p16^INK4A ^overexpression might specifically identify HPV infections that are biologically relevant in the carcinogenesis of head and neck squamous cell carcinomas and cervical carcinomas. However, it should be noted that expression of p16^INK4A ^is not limited to HPV-positive tumors, and the use of this marker alone as an indicator of biologically relevant HPV infections inevitably entails the risk of including some HPV negative p16^INK4A ^positive results [45]. In the present study, no significant correlation could be found between p16^INK4A ^expression and the presence of an HPV infection. This might be explained by the fact that p16^INK4A ^is aberrantly methylated in about 40% of sporadic CRC and is significantly correlated with loss of tumor suppressor function [47]. In the current study 26% of analyzed samples did not show p16^INK4A ^expression, which is in agreement with the literature. Few studies have investigated the relationship between p16^INK4A ^expression and colorectal adenocarcinoma. The reported frequency of p16^INK4A ^protein expression in CRC varies from 17 to 99%, with the majority of investigations showing p16^INK4A ^expression in more than two third of CRC [48]. In this study, we noted that p16^INK4A ^protein was expressed in 74% of the colorectal adenocarcinomas and more than half (n = 38) showed a high level of p16^INK4A ^expression. P16 is a nucleoprotein; the presence of staining in both the nuclei and the cytoplasm supports the finding that p16 gene is overexpressed. The change in subcellular location of the overexpressed nucleoprotein might account for its role in CRC carcinogenesis. The mechanism inducing the p16^INK4A ^overexpression is probably different from promoter methylation and could presumably result from a compensatory response to cell cycle deregulation [48]. CDK4 overexpression could be the initial event leading to a reactive overexpression of p16^INK4A ^and to a break in the G1-S transition through pRb phosphorylation [49].

No significant differences in clinical and pathological variables were found between CRC samples expressing p16^INK4A ^and those not expressing p16^INK4A^, except for location. In accordance with others [47,48] p16^INK4A ^protein expression was more often seen in the distal colon and the rectum. In addition, there was a trend towards an association between strong p16^INK4A ^expression and stage II and III tumors.

The prognostic role of p16^INK4A ^protein has been investigated in five studies. Three studies noted that p16^INK4A ^expression was associated with poorer survival [48-50]. In accordance to Norrie et al. [51] and Tada et al. [52], we found no relationship between p16^INK4A ^expression and patient survival.

## Conclusions

In conclusion, the presence of oncogenic HPV DNA in a small cohort of CRC samples, with high risk HPV 16 as the most prevalent type, was confirmed in the present study. However, in order to suggest that HPV might be involved in colon cancer, viral DNA incorporation into the host genome needs to be demonstrated by in situ hybridization. Additionally, the presence or absence of HPV in a non-malignant control group and tumor adjacent tissue needs to be investigated in order to determine whether HPV is merely an epiphenomenon in CRC or rather a potential cofactor in the development of the disease.

In addition, contrary to what has been observed in head and neck squamous cell cancer and cancer of the uterine cervix, p16^INK4A ^does not seem to be a surrogate marker for an active HPV infection in CRC. Therefore, further functional analyses are necessary to elucidate the significance of the presence of HPV in CRC.

## Competing interests

The authors declare that they have no competing interests.

## Authors' contributions

VD designed the study, carried out the PCR and genotyping analysis, the IHC scoring, the statistical analysis and the interpretation of the results and drafted the manuscript. MB participated in the study design, set up the IHC scoring system, carried out the IHC scoring and helped to draft the manuscript. VMV participated in setting up the IHC scoring system carried out the IHC scoring and revised the manuscript. EVM participated in the IHC and supervised the scoring and revised the manuscript. CW participated in the PCR and genotyping analysis and VF supervised these analyses and revised the manuscript. PV provided tissue samples and coordinated sifting through the medical files. FL and JBV participated in the design and coordination of the study and helped to draft the manuscript. All authors read and approved the final manuscript.

## Pre-publication history

The pre-publication history for this paper can be accessed here:

http://www.biomedcentral.com/1471-2407/10/117/prepub
